# Exploring the emotional mechanism of consumer satisfaction in new energy vehicles: a dual-path model of intelligent and eco-friendly experiences

**DOI:** 10.3389/fpsyg.2024.1436494

**Published:** 2024-08-21

**Authors:** Shizhen Bai, Tao Sun, Hao He

**Affiliations:** School of Management, Harbin University of Commerce, Harbin, China

**Keywords:** emotional mechanism, consumer satisfaction, perceived usability, intelligent experience, eco-friendly experience

## Abstract

New energy vehicles (NEVs) are considered a crucial means of reducing travel costs, enhancing consumer experiences, and innovating services. This paper aims to categorize the functional experiences of NEVs into two types: intelligent experience and eco-friendly experience, using a dual-path model. By analyzing 118,648 text data from automotive information and service platforms, the relevant factors influencing consumer satisfaction are explored. The research findings reveal that intelligent experience has a significantly positive impact on consumer satisfaction, whereas eco-friendly experience has a significantly negative impact on consumer satisfaction. This suggests that new energy vehicle companies need technological innovation in infrastructure and range to enhance consumer satisfaction. Furthermore, the research confirms that, when facing the intelligent experience of new energy vehicles, consumers’ “rational” thinking plays a dominant role, and efficacy is an intermediate variable to enhance consumer satisfaction. On the other hand, when facing the eco-friendly experience of new energy vehicles, consumers’ “emotional” thinking predominates, and identity is an intermediate variable to enhance consumer satisfaction. Additionally, the moderating effect of perceived usability is examined. When faced with the intelligent experience of NEVs, consumers with high perceived usability are more likely to generate a sense of efficiency. In the case of the eco-friendly experience of NEVs, consumers with low perceived usability are more likely to experience a sense of identity.

## 1 Introduction

On the path to a low-carbon and zero-carbon future, breakthroughs in green low-carbon technologies, represented by NEVs, are highly anticipated. NEVs, with their clean and environmentally friendly advantages, have become an indispensable choice for consumers on the journey of car ownership. Policies proposing the ban on sales of traditional fuel vehicles by numerous countries and comprehensive electrification development strategies put forth by automotive manufacturers indicate that the global new energy vehicle industry, especially the electric vehicle sector, will encounter more development opportunities in the next decade (Liu et al., 2023). NEVs have created various values, such as reducing travel costs and enhancing consumer experiences (Li et al., 2023).

With the growing ecological awareness among people, consumer preferences for NEVs have become a significant trend in green consumption (Zhang et al., 2020). Existing literature on factors influencing consumer satisfaction typically discusses individual consumer characteristics (Li and Shao, 2023), product attributes (Yang and Qiao, 2023), external environments (Fan and Wang, 2022), and others. However, consumers may encounter unfavorable driving experiences while using NEVs, leading to strong dissatisfaction, as evidenced by consumers expressing their discontent through numerous negative reviews (Xiong and Shu, 2023). Additionally, the potential impact of subsidy policy changes on consumer attitudes toward car purchases after the decline in subsidies is also a subject of investigation (Yang et al., 2023). The former focuses on rational or objective evaluations of NEVs, while the latter reflects consumers’ emotional or subjective biases.

Previous scholars have analyzed the impact of intelligent attributes (Yu, 2020) and eco-friendly attributes of green products (Cheng et al., 2015) on consumer satisfaction. However, empirical research that comprehensively evaluates both intelligent and eco-friendly attributes in the context of NEVs is relatively scarce. Some scholars argue that environmental pollution can stimulate a sense of urgency and responsibility to protect the environment, thereby promoting green consumption (Fritsche and Häfner, 2012). People may reduce guilt toward the environment by purchasing and driving NEVs. However, existing literature provides contradictory evidence. Some scholars have found no correlation between environmental pollution and green consumption tendencies, even suggesting that the intelligent attributes of NEVs are the primary driver of consumer satisfaction (Li et al., 2023). These contradictory conclusions underscore the importance of in-depth exploration into the relationship between the attributes of new energy vehicles and consumer satisfaction.

By reviewing the existing literature, this study identifies the inconsistent conclusions mentioned above, revealing several key issues existing in the current body of research: (1) existing studies mainly focus on individual, discrete perceived values (such as positive emotions) and their impact on consumer satisfaction (Lin et al., 2020), lacking a comprehensive study that integrates perceived values into cognitive paths and categorizes them; and (2) previous literature has explored the promotion of consumer satisfaction from the perspectives of rational (cognitive) cognition (such as planned behavior theory, norm activation theory) (Li and Shao, 2023) and emotional responses from emotional (non-rational) cognition (such as reducing environmental pollution, protecting the environment) (Li et al., 2023). However, as perceived values lead to cognitive evaluations and emotional responses, a comprehensive perspective is needed for research. This paper, based on a dual-path model, explores the impact of intelligent and eco-friendly attributes of NEVs on consumer satisfaction, as well as their moderating and mediating mechanisms. The research aims to achieve the following objectives: (1) validate the promoting role of two types of functional experiences in enhancing consumer satisfaction, providing new theoretical foundations for improving consumer satisfaction; (2) based on two cognitive “thinking” processes, consider efficacy and identity as two emotional responses serving as mediating mechanisms for the promotion of consumer satisfaction by two types of functional experiences, with in-depth exploration of their contingency; and (3) use perceived usability to verify the boundary conditions of how the two types of functional experiences influence emotional responses.

## 2 Theoretical foundation and research hypotheses

### 2.1 The intelligent experience and eco-friendly experience of NEVs are associated with consumer satisfaction

Compared to traditional fuel vehicles, NEVs place greater emphasis on intelligent interaction with consumers and the use of clean energy in their functional design (Li et al., 2012), providing an opportunity to create sustainable value. Exploring NEVs from the perspective of consumer value perception is conducive to better applying technological innovations, enhancing consumer satisfaction, and promoting the widespread adoption of NEVs. The two key attributes of NEVs, namely intelligent attributes and eco-friendly attributes, hold significant importance for consumer satisfaction (Li et al., 2012). In contrast to traditional fuel vehicles, the intelligent attributes of NEVs focus on intelligent interaction between consumers and the vehicle, such as smart navigation (Gao et al., 2019; Peng et al., 2020), and data privacy security (Li et al., 2020). These features reflect the positive driving experience brought about by the diversified functionalities of NEVs. Simultaneously, their eco-friendly attributes manifest in low carbon emissions (Peng et al., 2020), intelligent charging (Bockarjova and Steg, 2014), providing consumers with a cleaner and cost-effective travel option.

Consumer expectations are formed based on their expectations of the vehicle’s performance and features before using NEVs. Subsequent satisfaction assessments are based on the comparison between these expectations and the actual experience, forming a psychological state of satisfaction. Cronin et al. (2000) found that service quality, service value, and satisfaction may be directly related to behavioral intentions. Borghi et al. (2023) analyzed 20,166 online reviews of hotel service robots using machine learning and natural language processing techniques, revealing improved customer satisfaction through the relationship between service robots and customers. Heinen et al. (2023) studied how smart charging can successfully reduce peak demand and enhance customer satisfaction. Kumar et al. (2023) explored the determinants of customer satisfaction using online reviews of a grocery mobile application, suggesting that managers should focus on factors such as the ordering experience, delivery personnel interaction, and payment experience to improve customer satisfaction. Li et al. (2023) conducted sentiment analysis and text mining on 85,306 online reviews of eight types of typical green products in four areas on the JD e-commerce platform, finding variations in consumer preferences and concerns for different attributes of green products. Ramón (2023) found that customer attention and food quality are often used to define restaurant experiences, and the study revealed that the factors identified varied in their impact on customer satisfaction depending on the restaurant rating. Wu and Huo (2023) explored the relationship between service robots, consumer service experience perception, and consumer satisfaction, indicating a positive impact of introducing service robots in hotels on consumer satisfaction, with perceived warmth and competence mediating this impact (Wu and Huo, 2023). Therefore, this study proposes the following hypotheses:

*H1a*: The intelligent experience of NEVs has a positive impact on consumer satisfaction.

*H1b*: The eco-friendly experience of NEVs has a positive impact on consumer satisfaction.

### 2.2 Emotional responses

Emotional responses represent the psychological processes and accompanying physiological reactions that individuals have to external stimuli, encompassing various sensations, thoughts, and behaviors (Berger, 2011).

In recent years, scholars have conducted in-depth research on the relationship between emotional responses and consumer satisfaction, covering multiple consumer scenarios. Previous research indicates that consumer emotional responses significantly influence subsequent satisfaction experiences, including emotional responses when facing uncertainty, which are directly related to subsequent satisfaction experiences (Xu and Guo, 2023). Guo et al. (2021) focused on apology commitment-type management feedback strategies, examining their impact on customer satisfaction and highlighting the importance of emotional regulation effects. Wang et al. (2023) studied the impact of marketing orientation on consumer regret emotions in the context of overconsumption, revealing that marketing orientation affects subsequent satisfaction experiences through its influence on responsibility attribution and self-control. Fei et al. (2021) delved into the persuasive effects of awe emotions, finding that awe emotions can influence individuals’ cognitive and emotional responses, subsequently impacting satisfaction.

This study chooses to investigate emotional responses for two reasons: (1) emotional responses, different from behavioral responses, cover non-rational factors such as emotions, intuitions, cognitive and evaluative rational factors (Septianto et al., 2023), providing a better explanation for the complex psychological mechanisms that drive consumer satisfaction promoted by functional experiences; (2) emotional responses are related to consumer behavioral responses, serving as a fundamental mechanism for consumers to react to the product attributes of NEVs, prompting consumers to adopt environmentally friendly behaviors favoring new energy vehicle consumption when facing environmental pollution and the call for “dual-carbon,” making it one of the essential psychological factors to enhance consumer satisfaction (Mano and Oliver, 1993).

This study integrates efficacy and identity, two different orientations and valence of emotional responses, into the research framework. Efficacy represents the emotional response to intelligent attributes, while identity represents the emotional response to eco-friendly attributes. Since intelligent attributes involve functions such as data privacy security, autonomous driving, and smart navigation, consumers’ perception of these functions triggers efficacy, enhancing satisfaction. On the other hand, eco-friendly attributes involve the advantages of NEVs in low carbon emissions, smart charging, etc., and this perception triggers identity, prompting consumers to choose such vehicles more satisfactorily. Therefore, the study proposes the following hypotheses:

*H2a*: The sense of efficiency plays a mediating role between intelligent experience and consumer satisfaction.

*H2b*: The sense of identity acts as a mediating factor between eco-friendly experience and consumer satisfaction.

### 2.3 Perceived usability

Introducing Davis’s (1989) Technology Acceptance Model, which aims to explain the determining factors of users’ decisions to accept information systems. The core idea includes two major determining factors: perceived usefulness, i.e., the extent to which a person believes that using the system will enhance job performance, and perceived ease of use, i.e., the ease with which a person believes they can use the system (Davis, 1989).

**Figure 1:**
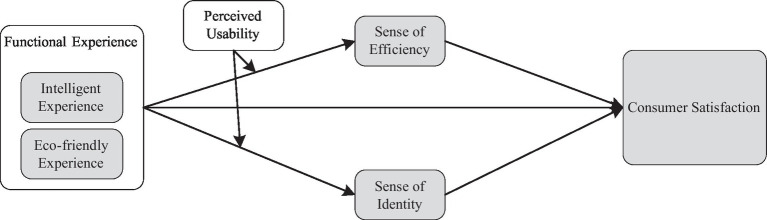
Research framework.

Devaraj et al. (2002) found that perceived usefulness and perceived ease of use are critical determinants of consumer attitudes and indirectly impact consumer satisfaction through channel preferences. De Guinea et al. (2014) found that, in high frustration situations, neural physiological memory load has a negative impact on perceived ease of use, demonstrating the importance of emotional perception in regulating the impact of neurophysiological states on behavioral beliefs. Lee et al. (2012) found that the emotions displayed by users on Facebook activity pages significantly impact the perceived ease of use and perceived enjoyment of social media marketing (Lee et al., 2012). Gefen et al. (2003) highlighted the significance of consumer trust, as with widely accepted TAM antecedents, such as perceived usefulness and perceived ease of use, for electronic commerce. Partala and Saari (2015) found that perceived usefulness, satisfaction of psychological needs, and negative emotions significantly affect the overall perceived value of user experiences in the most influential user experiences. Pelegrín-Borondo et al. (2017) established a cognitive-emotional-normative model, confirming that emotional and normative factors have the greatest impact on the acceptance of new technology, and perceived usability has a positive impact on the generation of emotions, specifically on mood. In this study, perceived usability reflects the efficacy and identity generated by consumers when driving NEVs, thereby enhancing satisfaction. Therefore, the regulatory role of perceived usability in the respective functional experiences is crucial (Figure 1). In summary, this study proposes the following hypotheses:

*H3a*: Perceived usability plays a moderating role between intelligent experience and the sense of efficiency.

*H3b*: Perceived usability plays a moderating role between eco-friendly experience and the sense of identity.

## 3 Data collection and processing

### 3.1 Sample selection

In the context of the global energy transition, the NEVs industry emerges as a key driver for sustainable development, playing a crucial role in both the Chinese and global markets. NEVs are gradually replacing traditional fuel vehicles, a trend that cannot be ignored. Undoubtedly, China establishes itself as a global powerhouse in the new energy vehicle sector. NEVs in the Chinese market consistently top global production and sales charts, garnering significant favor from consumers at home and abroad. This underscores the importance of exploring various factors influencing consumer satisfaction, including intelligent experience, eco-friendly experience, perceived usability, sense of efficiency, sense of identity, consumer satisfaction, and functional experiences.

Notably, on January 31, 2024, the renowned domestic new energy vehicle business intelligence data service provider, QuestAuto, released the “2023 New Energy Vehicle Market Report.” The report indicates that the new energy vehicle market experienced a significant surge in 2023, not only witnessing a substantial increase in sales but also a remarkable rise in active increments. Chinese domestic brands, in particular, presented a flourishing scenario of diversity.

In this study, we have selected NEVs as our research samples, aiming to delve into the impact of innovative and environmentally friendly vehicle models on consumer satisfaction. Given the leading position of NEVs in technology, environmental protection, and sustainability, the academic community has paid special attention to this field. NEVs not only represent a technological upgrade from traditional fuel models but also attract widespread attention due to their unique functional experiences. Furthermore, through the analysis of user reviews, we can capture the genuine feelings and opinions of consumers, providing a unique perspective for a deeper understanding of the intrinsic drivers of satisfaction. Finally, studying NEVs helps understand consumers’ psychological reactions when facing innovative products, offering targeted market strategies and product design recommendations for automotive manufacturers.

### 3.2 Data sources and explanation

This study utilized online reviews of 32 NEVs from three prominent automotive information and service platforms as the primary data source. These three platforms are “Autohome,” “PacAuto,” and “Yiche,” each enjoying outstanding recognition in the automotive industry. They offer users timely and comprehensive services such as automotive user reviews, vehicle information, and model evaluations, providing crucial references and support for consumer car purchasing decisions.

To obtain data, a Python based scraping program is employed in a targeted manner to extract user comments about 32 NEVs from the three platforms. During the data collection process, a specific collection template was used, and by November 25, 2023, a total of 118,648 data items, including user ID, comment text, comment date, rating, and vehicle model, were successfully collected. These data were stored in CSV format, providing a comprehensive and robust foundation for subsequent analysis.

### 3.3 Data preprocessing

#### 3.3.1 Noise data eliminating

In the data collection process, due to the substantial workload and frequent operations, some duplicated, incorrect, invalid, and meaningless data inevitably occurred, resulting in non-standardized data presentation on the page. To ensure the accuracy of subsequent analyses, this study referred to previous experiences (Wang et al., 2022) and meticulously cleaned the 118,648 original comments, eliminating duplicates, invalid reviews unrelated to the product, comments composed of emoticons and special characters, and low-reference-value reviews. After the data elimination process, this study obtained a total of 86,502 valid new energy vehicle comments.

Typically, consumer comments on e-commerce platforms are more colloquial and lack standardized structures or uniform formats. Therefore, before proceeding with further analysis, the data required additional processing, including segmentation and stop-word removal, to convert it into a machine-readable form. Referring to previous relevant studies (Berger et al., 2020), this study, in the cleaned comments, utilized authoritative Chinese and international *jieba* segmentation and stop-word tables for segmentation operations and removal of stop words.

#### 3.3.2 STM topic model and LIWC

**Figure 2:**
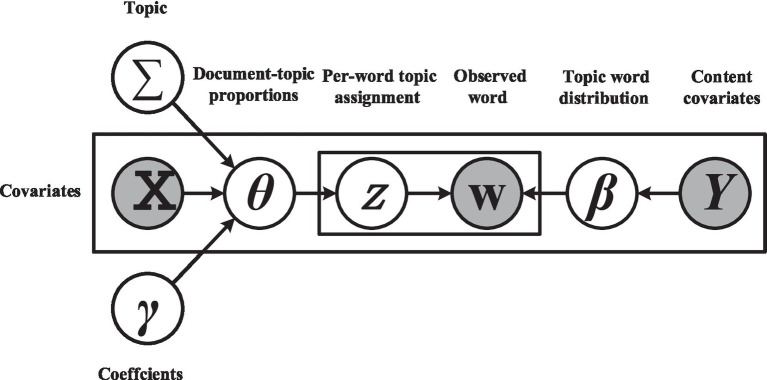
STM schematic diagram.

STM (Structural Topic Model) is a topic model used for text data analysis, exhibiting outstanding performance in text topic modeling. STM can effectively capture structural relationships between documents. In this study, we used the comment time and rating of new energy vehicle comments as popularity covariates, inputting them into STM for topic extraction. After experimental validation, when the number of topics was set to 27, STM demonstrated the optimal extraction performance, showing good semantic consistency and exclusivity in training results. We labeled each topic based on its most representative words, combined with domain knowledge (Tsui et al., 2010; Chuah et al., 2021), and summarized the types of topics. Subsequently, we selected topics related to intelligent and environmentally friendly attributes to explore consumer functional experiences as variables validating increased satisfaction. Additionally, we selected topics related to perceived usability values (Wolf and Seebauer, 2014; Liu and Park, 2015) to uncover consumer perceptual tendencies as variables validating the relationship between functional experiences and emotional responses (Figure 2).

This study is based on the Python language and the LIWC (Linguistic Inquiry and Word Count), conducting in-depth analysis of language proficiency and preferences among different categories in the text. LIWC reads a text, the text analysis module then compares each word in the text against a user-defined dictionary. The dictionary identifies which words are associated with which psychologically-relevant categories. After read and accounted for all words in a given text, it calculates the percentage of total words that match each of the dictionary categories. We employed LIWC for a detailed examination of emotional and psychological factors. Through text feature extraction, we calculated the proportions of various textual features, including the percentage of multifunctional words and positive emotion words. Among these multifunctional words, we further extracted the sense of efficiency, while in positive emotion words, we extracted the sense of identity. These were used as intermediary variables to validate the relationship between functional experiences and consumer satisfaction.

In summary, this study utilizes the proportions of topics related to functional experiences and perceived values, the proportions of text features analyzed by LIWC, and user ratings as the basis for analysis.

## 4 Results and discussion

### 4.1 Main effect tests

To begin with, we employ SPSS 25.0 for descriptive statistics and correlation analysis on various variables, and the detailed results can be found in Table 1. There exists a significant correlation (*p* < 0.05) among intelligent experience, eco-friendly experience, perceived usability, sense of efficiency, sense of identity, and consumer satisfaction.

**Table 1:** Descriptive statistics and correlation analysis of variables.

	*M*	SD	Intelligent experience	Eco-friendly experience	Perceived usability	Sense of efficiency	Sense of identity	Consumer satisfaction
Intelligent experience	0.04	0.05	1					
Eco-friendly experience	0.03	0.05	−0.047**	1				
Perceived usability	0.03	0.04	0.050**	0.312**	1			
Sense of efficiency	0.10	0.03	−0.020**	0.074**	0.093**	1		
Sense of identity	0.04	0.01	−0.124**	−0.040**	0.092**	−0.128**	1	
Consumer satisfaction	4.54	0.44	0.090**	−0.057**	0.01**	0.059**	−0.015**	1

Furthermore, linear regression analyses were conducted to examine the relationships between intelligent experience, eco-friendly experience, and consumer satisfaction. The results, as presented in Table 2, reveal a significant positive impact of intelligent experience on consumer satisfaction (*p* < 0.001, *β* = 0.090). Therefore, hypothesis H1a is supported, indicating a close association between technological innovation and the application of intelligent features in NEVs and consumer satisfaction.

**Table 2:** Linear regression analysis.

	Unstandardized coefficient		Standardized coefficient	*t*	Significance
	*B*	Standard deviation	Beta		
(Intercept)	4.504	0.002		2331.211	0.000
Intelligent experience	0.818	0.031	0.090	26.550	0.000
(Intercept)	4.555	0.002		2417.092	0.000
Eco-friendly experience	−0.563	0.033	−0.057	−16.935	0.000

Firstly, the technological innovations in intelligent assisted driving and voice recognition contribute to the convenience, safety, and comfort of the driving process, significantly enhancing overall consumer satisfaction. This may be attributed to the personalized driving experiences facilitated by intelligent technologies (Cui et al., 2019), further reinforcing user acceptance and satisfaction with NEVs. Secondly, the convenience and safety improvements brought about by intelligent experience not only meet consumers’ expectations for vehicle performance but also strengthen their positive perceptions of NEVs. Through a more intelligent driving experience, consumers are more likely to establish trust in NEVs, thereby promoting an increase in their satisfaction.

**Figure 3:**
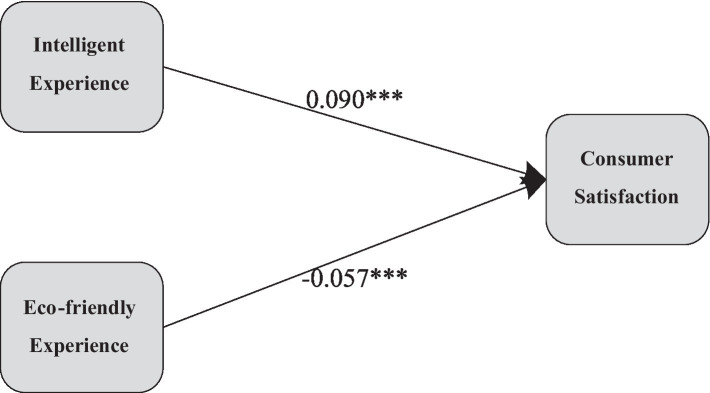
Pathway diagram of intelligent experience and eco-friendly experience.

The eco-friendly experience (*p* < 0.001, *β* = −0.057) exhibits a significant negative impact on consumer satisfaction, thus rejecting the hypothesis H1b. The environmentally friendly attributes of NEVs may, under specific conditions, result in a negative influence on consumer satisfaction. Firstly, the eco-friendly attributes of NEVs have a detrimental effect on the driving experience for consumers under low-temperature conditions, such as concerns related to battery range and travel costs (Whitehead and Wicker, 2018). This could lead to inconvenience and additional expenses for consumers in cold environments, consequently diminishing their overall satisfaction with NEVs. Secondly, in cold conditions, the increased operational costs of NEVs may not solely result from a decline in battery range but also encompass additional energy consumption by the heating system. To maintain a comfortable interior temperature, vehicles may require additional power supply for heating devices, thereby diminishing the available energy in the battery and consequently affecting the overall range performance. These factors contribute to a reduced practicality of electric vehicles in specific environments, subsequently amplifying consumer misunderstandings and dissatisfaction. The intelligent experience of NEVs is compromised under such circumstances, highlighting the intricate interplay between eco-friendly experiences, perceived usability, sense of efficiency, and consumer satisfaction (Figure 3).

### 4.2 Mediation effect tests

To examine the mediating role of emotional responses in the impact of functional experiences on consumer satisfaction, this study employs the Bootstrap method for analysis, with a sample size of 5,000. The analysis was conducted using Model 4 from the PROCESS macro program in SPSS, with a 95% confidence interval. Tables 3, 4 respectively present the results of the mediating effects of the sense of efficiency and the sense of identity.

**Table 3:** Mediation analysis of efficacy perception.

	Effect	SE	*t*	*p*-value	LLCI	ULCI
Total effect	0.818	0.031	26.550	0.000	0.758	0.879
Direct effect	0.829	0.031	26.945	0.000	0.769	0.889
Indirect effect	−0.011	0.002			−0.014	−0.007

**Table 4:** Mediation analysis of identity perception.

	Effect	SE	*t*	*p*-value	LLCI	ULCI
Total effect	−0.563	0.033	−16.935	0.000	−0.628	−0.498
Direct effect	−0.570	0.033	−17.125	0.000	−0.635	−0.504
Indirect effect	0.007	0.002			0.004	0.010

**Figure 4:**
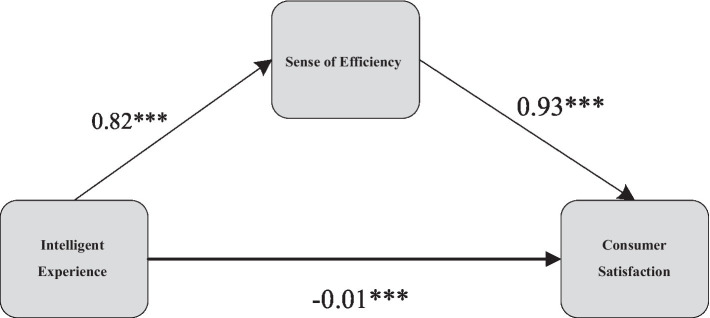
Mediation path diagram of efficacy perception in the relationship between intelligent attributes and consumer satisfaction.

According to the research results presented in Table 3, introducing the mediating variable, the sense of efficiency, the total effect showed significance (*p* < 0.001, *β* = 0.818). Upon closer inspection, both the direct effect (*p* < 0.001, *β* = 0.031) and the mediating effect of the sense of efficiency are significant (*β* = −0.011, SE = 0.002, 95%CI = [−0.014, −0.007]), providing clear support for the validity of hypothesis H2a. The detailed diagram illustrating the mediating pathways can be found in Figure 4. This series of findings strongly indicates that the sense of efficiency plays a crucial mediating role in the impact of intelligent experiences on consumer satisfaction.

When consumers driving NEVs perceive intelligent attributes, a “pleasure” thought pattern dominates their emotional responses, triggering a sense of efficiency. This emotional response, through partial mediation, further elevates the level of consumer satisfaction. The joy and convenience brought about by intelligent experiences trigger the “pleasure” emotional response (Kim and Park, 2019), which, through the mediating role of the sense of efficiency, enhances overall satisfaction with the experience. Therefore, we can conclude that the sense of efficiency plays a critical mediating role in the impact of intelligent experiences on consumer satisfaction.

The sense of efficiency is a psychological perception of individuals toward the abundant and powerful functions of NEVs, closely linked to the “pleasure” thought pattern. When consumers perceive intelligent attributes, their thought patterns are guided toward a state of enjoyment and pleasure (Alalwan, 2020), resulting in a sense of efficiency. This emotional cognition, through partial mediation, further increases overall satisfaction levels. The joy and convenience brought about by intelligent experiences act as triggering factors for the “pleasure” emotional response, and the sense of efficiency, as a mediator, profoundly connects the relationship between emotional responses and satisfaction.

**Figure 5:**
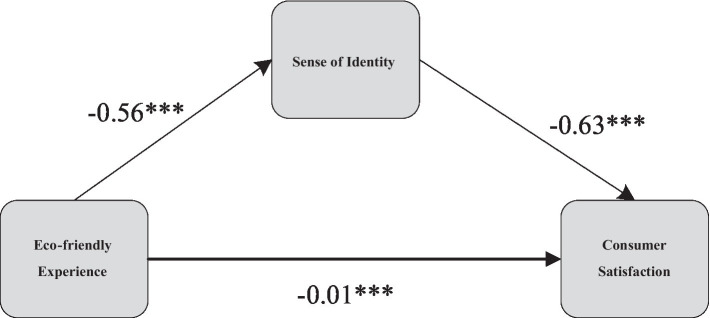
Mediation path diagram of identity perception in the relationship between eco-friendly attributes and consumer satisfaction.

According to the results presented in Table 4, after incorporating the mediating variable, the sense of identity, into the model, the total effect showed significance (*p* < 0.001, *β* = −0.563). Further observation reveals that both the direct effect (*p* < 0.001, *β* = −0.570) and the mediating effect of the sense of identity were significant (*β* = −0.007, SE = 0.002, 95%CI = [0.004, 0.010]), providing robust support for hypothesis H2b. The corresponding diagram illustrating the mediating pathways can be found in Figure 5. This series of results clearly indicates that in the impact of eco-friendly experiences on consumer satisfaction, the sense of identity plays a partial mediating role.

When consumers driving NEVs perceive eco-friendly attributes, their thought patterns are primarily influenced by “compliance,” triggering emotional responses and ultimately generating a sense of identity (Kunkel et al., 2021). This emotional response, through partial mediation, further enhances consumer satisfaction. The mediating role of the sense of identity in eco-friendly experiences further emphasizes that in the market promotion of NEVs, eco-friendly attributes serve not only to meet societal expectations but also, by triggering a sense of identity, enhance overall consumer satisfaction. Therefore, the sense of identity plays a positive mediating role in the impact of eco-friendly experiences on consumer satisfaction.

The thought pattern of “compliance” triggered by eco-friendly attributes generates a sense of identity among consumers, leading them to more positively experience eco-friendly attributes (Carter and Gilovich, 2012). The sense of identity, as an emotional cognition, through partial mediation, further influences consumer satisfaction. Therefore, the sense of identity not only plays a role in meeting societal expectations but also, on an emotional level, by strengthening consumer identification with eco-friendly experiences, enhances overall satisfaction levels.

### 4.3 Moderation effect tests

To examine the moderating effect of perceived usability in the mediating model involving functional experiences, emotional responses, and consumer satisfaction, this study utilized the Bootstrap method for analysis with a sample size of 5,000. The analysis, conducted at a 95% confidence interval, employed Model 7 from the PROCESS macro program in SPSS. Tables 5, 6 respectively display the results of the moderating effect of perceived usability.

**Table 5:** Sense of efficiency of total effects, direct effects, and indirect effects (bootstrap = 5,000).

Sense of efficiency	Effect	Boot SE	Boot LLCI	Boot ULCI
Total effect	0.009	0.002	0.004	0.014
Direct effect	−0.015	0.002	−0.019	−0.011
Indirect effect	−0.046	0.004	−0.053	−0.039

**Table 6:** Sense of identity of total effects, direct effects, and indirect effects (bootstrap = 5,000).

Sense of identity	Effect	Boot SE	Boot LLCI	Boot ULCI
Total effect	0.011	0.003	0.006	0.017
Direct effect	0.005	0.001	0.003	0.008
Indirect effect	−0.003	0.001	−0.006	−0.002

#### 4.3.1 Moderating effect of perceived usability on the relationship between smart experience and efficiency perception

As shown in Table 7, smart experience significantly negatively influences efficiency perception (*p* < 0.001, *β* = −0.016), while efficiency perception has a significant positive impact on consumer satisfaction (*p* < 0.001, *β* = 0.934). Notably, in this context, smart experience also exhibits a significant positive influence on consumer satisfaction (*p* < 0.001, *β* = 0.829), indicating that efficiency perception acts as a partial mediator between smart experience and consumer satisfaction. This finding emphasizes the crucial role of efficiency perception in the overall formation process of consumer satisfaction.

**Table 7:** Regression analysis results of relevant variables in the moderated mediation model.

Variable	Sense of efficiency		Sense of identity		Consumer satisfaction	
	*β*	*p*-value	β	*p*	β	*p*-value
Intelligent experience	−0.016	0.000			0.829	0.000
Sense of efficiency					0.934	0.000
Intelligent experience* sense of efficiency	−0.778	0.000				
Eco-friendly experience			−0.008	0.000	−0.570	0.000
Sense of identity					−0.629	0.000
Eco-friendly experience* sense of identity			0.314	0.000		

In Table 5, perceived usability (*p* < 0.001, *β* = −0.015, 95%CI = [−0.019, −0.011]) also exhibits significance in terms of mediating effects. The results reveal that the interaction term between smart experience and perceived usability significantly affects efficiency perception (*p* < 0.001, *β* = −0.778). Further simple slope analysis reveals a significant positive impact of smart experience on efficiency perception in individuals with low perceived usability (*p* < 0.001, *β* = 0.009, 95%CI = [0.004, 0.014]). However, in individuals with high perceived usability, smart experience significantly negatively influences efficiency perception (*p* < 0.001, *β* = −0.049, 95%CI = [−0.055, −0.043]). This result suggests that perceived usability plays a significant moderating role in the relationship between smart experience and efficiency perception.

A more in-depth analysis of indirect effects indicates that, for individuals with low perceived usability, smart experience further promotes consumer satisfaction by enhancing the positive impact on efficiency perception (*p* < 0.001, *β* = 0.009, 95%CI = [0.004, 0.014]). Conversely, in individuals with high perceived usability, the negative impact of smart experience on efficiency perception slows down the positive effect on consumer satisfaction (*p* < 0.001, *β* = −0.046, 95%CI = [−0.053, −0.039]). The significant difference in indirect effects between high and low levels is noteworthy, with a difference of −0.054, 95%CI = [−0.064, −0.046], *p* < 0.001, confirming the validity of H3a. This further validates the moderating role of perceived usability in the relationship between smart experience and consumer satisfaction.

On the one hand, individuals with low perceived usability may have lower adaptation levels when facing new technologies. Therefore, they are more likely to generate positive experiences with the innovative and convenient aspects of the intelligent attributes of NEVs. This positive experience may stimulate their sense of efficiency, thereby enhancing overall satisfaction. These individuals may be more willing to accept and attribute high value to the positive effects brought about by intelligent experiences because it may provide them with more learning and growth opportunities (Thompson et al., 2005), subsequently elevating their recognition of the intelligent attributes of NEVs. Conversely, for individuals with high perceived usability, who have already established expectations for highly usable technology, they may hold relatively high standards for the performance and convenience of these technologies. When the intelligent attributes of NEVs fail to meet these high expectations, it may lead to a decrease in the sense of efficiency, thus slowing down the positive impact on overall satisfaction. This group may be more sensitive to the shortcomings of the technology because they have already established higher expectations in terms of usability.

**Figure 6:**
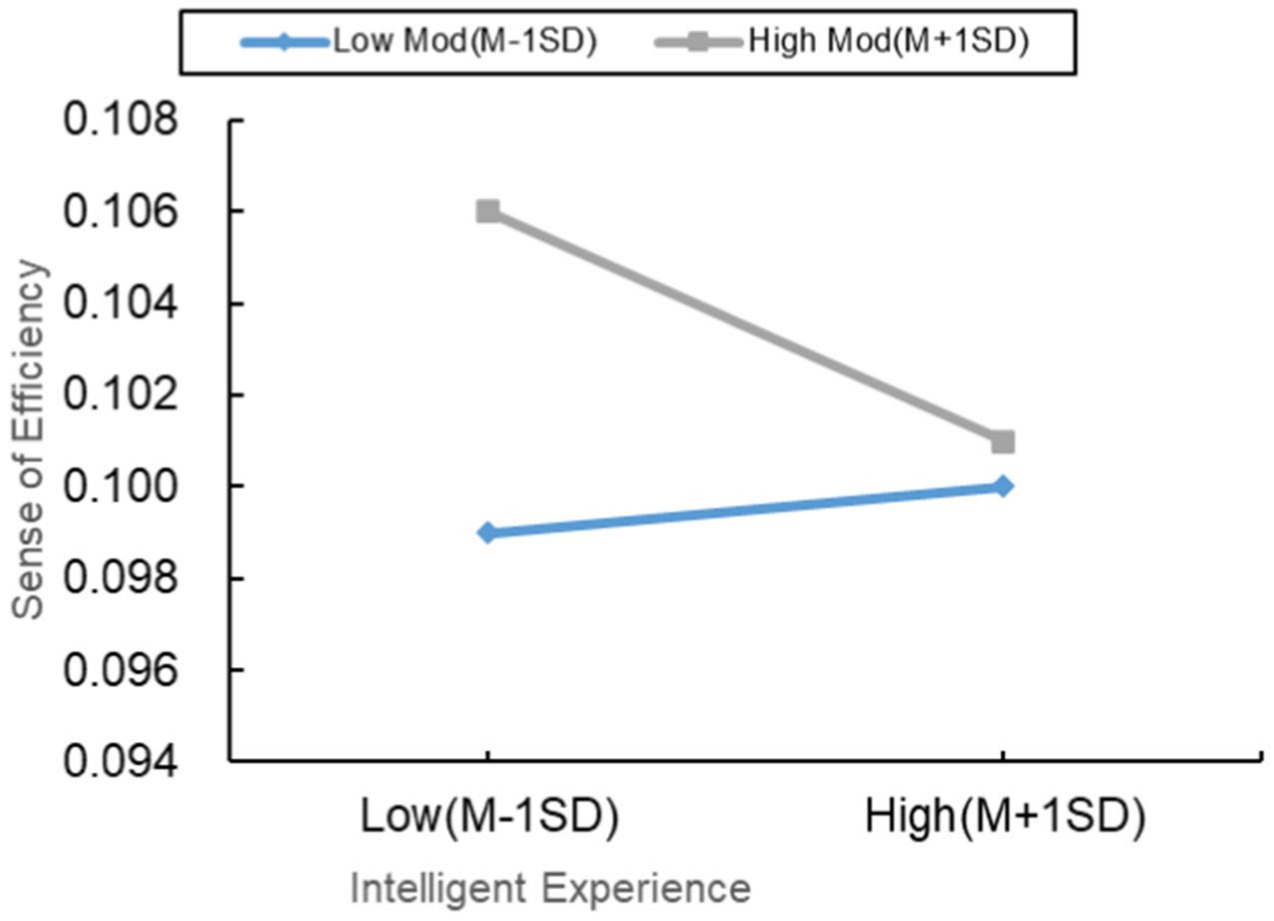
Moderating effect of perceived usability on the relationship between intelligent experience and efficacy perception.

On the other hand, individuals with high perceived usability may have higher expectations for the functionality and operation of NEVs. Therefore, if the intelligent experience fails to meet these expectations, it may result in a decreased sense of efficiency, thereby reducing overall satisfaction (Hong et al., 2017). This suggests that, for this group, intelligent experiences need to align with their high expectations (Belanche et al., 2021) to ensure that their overall evaluation of the intelligent attributes of NEVs remains positive. In contrast, for individuals with low perceived usability, their expectations for new energy vehicle technology may be relatively low. Even if the sense of efficiency from the intelligent experience is limited, they may still generate positive satisfaction with the vehicle. This could be because they place more emphasis on the basic functionalities of NEVs, and their requirements for advanced technology are relatively low, making them more easily satisfied. The moderating effect of perceived usability on the sense of efficiency is illustrated in Figure 6.

#### 4.3.2 Moderating effect of perceived usability on the relationship between environmental experience and identity perception

As shown in Table 7, environmental experience significantly negatively influences identity perception (*p* < 0.001, *β* = −0.008), while identity perception has a significant negative impact on consumer satisfaction (*p* < 0.001, *β* = −0.629). Importantly, in this context, environmental experience has a significantly positive influence on consumer satisfaction (*p* < 0.001, *β* = −0.570), indicating that identity perception acts as a partial mediator in this process. This finding emphasizes the crucial mediating role of identity perception in the relationship between environmental experience and consumer satisfaction.

As presented in Table 6, perceived usability (*p* < 0.001, *β* = 0.005, 95%CI = [0.003, 0.008]) also shows significance in terms of mediating effects. The results reveal that the interaction term between environmental experience and perceived usability significantly affects identity perception (*p* < 0.001, *β* = 0.314). Further simple slope analysis indicates a significant positive impact of environmental experience on identity perception in individuals with low perceived usability (*p* < 0.001, *β* = −0.018, 95%CI = [−0.021, −0.016]). However, in individuals with high perceived usability, environmental experience significantly negatively influences identity perception (*p* < 0.001, *β* = 0.005, 95%CI = [0.003, 0.007]). This result suggests that perceived usability plays a significant moderating role in the relationship between environmental experience and identity perception.

Further indirect effects analysis reveals that, for individuals with low perceived usability, environmental experience further promotes consumer satisfaction by enhancing the positive impact on identity perception (*p* < 0.001, *β* = 0.011, 95%CI = [0.006, 0.017]). However, for individuals with high perceived usability, the negative impact of environmental experience on identity perception leads to a decrease, weakening the positive impact on consumer satisfaction (*p* < 0.001, *β* = −0.003, 95%CI = [−0.006, −0.002]). The significant difference in indirect effects between high and low levels is noteworthy, with a difference of −0.015, 95%CI = [−0.022, −0.008], *p* < 0.001, further confirming the moderating role of perceived usability in the relationship between environmental experience and consumer satisfaction. In summary, assuming H3b is valid, perceived usability plays a moderating role in the relationship between environmental experience and consumer satisfaction (Table 8).

**Table 8:** Summary of hypothesis tests.

Hypotheses	Support
H1a: Perceived intelligent attributes of new energy vehicles have a positive impact on consumer satisfaction.	Yes
H1b: Perceived eco-friendly attributes of new energy vehicles have a positive impact on consumer satisfaction.	No
H2a: Efficacy mediates the positive impact of perceived intelligent attributes on consumer satisfaction.	Yes
H2b: Identity mediates the positive impact of perceived eco-friendly attributes on consumer satisfaction.	Yes
H3a: Perceived usability moderates the relationship between functional experiences triggered by intelligent attributes and efficacy.	Yes
H3b: Perceived usability moderates the relationship between functional experiences triggered by eco-friendly attributes and identity.	Yes

Firstly, for individuals with lower perceived usability, they are more likely to integrate the eco-friendly experience into their positive perception of NEVs (Gulzari et al., 2022), as they adapt more easily to the new environmental attributes. In their car purchasing decisions, these individuals place greater emphasis on the eco-friendly performance of NEVs, thereby increasing their satisfaction. Through the moderation of perceived usability, the eco-friendly experience has a positive impact on consumer satisfaction. Conversely, for individuals with higher perceived usability, they may pay more attention to other aspects of NEVs, such as performance or appearance, potentially having a negative impact on their sense of identity with the eco-friendly experience (Prebensen and Xie, 2017). This may be because, in the car purchasing decisions of these individuals, the importance of eco-friendly attributes is relatively lower compared to other factors. In this scenario, the negative impact of the eco-friendly experience on the sense of identity is weakened, contributing to an overall enhancement of satisfaction.

**Figure 7:**
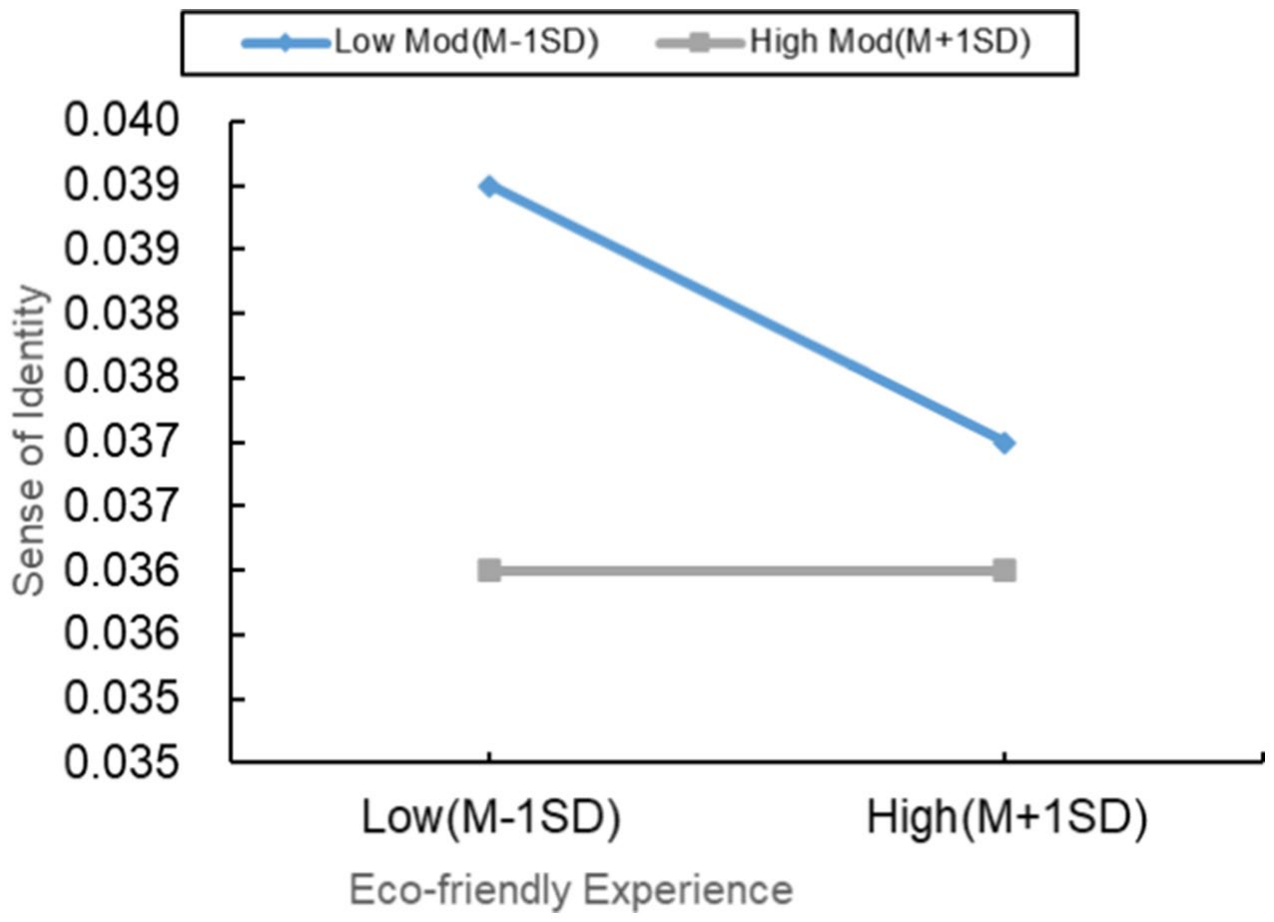
Moderating effect of perceived usability on the relationship between intelligent experience and efficacy perception.

On the other hand, individuals’ perceptions of eco-friendly experience, sense of identity, and perceived usability may vary due to differences in culture, education level, and life experiences (Sun et al., 2020). For example, in a cultural context emphasizing environmental ideals, the role of perceived usability may be more pronounced. Additionally, individuals’ education levels and life experiences may influence the importance they place on the eco-friendly attributes of NEVs. Therefore, when perceived usability is lower, individuals may be more willing to accept and identify with the eco-friendly experience. Conversely, when perceived usability is higher, they may prioritize other aspects, triggering the occurrence of moderation effects. The moderating effect of perceived usability on the sense of identity is depicted in Figure 7.

## 5 Research conclusion

In the face of technological constraints, this study investigates which attributes of NEVs can capture consumer attention and subsequently enhance consumer satisfaction. Initially, our focus centers on the relationship between functional experiences of NEVs and consumer satisfaction, examining the impact of different types of functional experiences on the psychological mechanisms underlying consumer satisfaction. Subsequently, we explore how two categories of functional experiences, guided by dominant intelligent and affective attributes respectively, activate distinct emotional responses through cognitive pathways to enhance consumer satisfaction. The conclusions affirm that regardless of the type of functional experience, it significantly influences consumer satisfaction with NEVs. However, different types of functional experiences exert their impact on consumer satisfaction through divergent cognitive processes and emotional responses.

Specifically, this paper elucidates the differential effects of intelligent experience and eco-friendly experience of new energy vehicles (NEVs) on consumer satisfaction. Firstly, the intelligent experience of NEVs has a significant positive impact on consumer satisfaction. This positive effect is primarily driven by the sense of efficiency rather than the sense of identity, as the intelligent experience focuses more on the hedonic value and enjoyment of NEVs. Secondly, the eco-friendly experience of NEVs has a significant negative impact on consumer satisfaction. This negative effect is primarily driven by the sense of identity rather than the sense of efficiency, as the eco-friendly experience emphasizes the emotional value and recognition of NEVs, leading to varying levels of perceived value (González-Mansilla et al., 2019). These findings help deepen the understanding of the boundary effects of different functional experiences on consumer satisfaction. Furthermore, when consumers experience the intelligent attributes of NEVs, their “cognitive” pathway is dominant, triggering a sense of efficiency and creating a psychological “hedonic” mechanism (Wei et al., 2013). Conversely, when consumers experience the eco-friendly attributes of NEVs, their “affective” pathway takes precedence, inducing a sense of identity and generating a psychological “conformity” mechanism (Qiao et al., 2023), thereby enhancing consumer satisfaction. The hedonic value of the intelligent experience of NEVs, through the enhancement of the sense of efficiency, primarily influences the consumers’ “cognitive” pathway, leading to positive emotional responses and high satisfaction. In contrast, the eco-friendly experience of NEVs, through the enhancement of the sense of identity, primarily influences the consumers’ “affective” pathway. Although it gains moral recognition, it may lead to negative emotional responses and lower satisfaction in actual usage.

Additionally, the study confirms that the impact of functional experiences on emotional responses is moderated by perceived usability. Specifically, perceived usability plays a crucial role in the emotional responses elicited by intelligent experience and eco-friendly experience. For intelligent experience, perceived usability moderates the effect of intelligent experience on the sense of efficiency. When consumers perceive the intelligent features of NEVs as easy to use, their sense of efficiency is significantly enhanced, triggering positive emotional responses and subsequently increasing consumer satisfaction. The high-tech attributes and convenience of the intelligent experience, through enhanced usability, elevate consumers’ enjoyment and satisfaction during use, ultimately boosting overall satisfaction. Conversely, for eco-friendly experience, perceived usability moderates the effect of eco-friendly experience on the sense of identity. When consumers perceive the eco-friendly features of NEVs as easy to use, their sense of identity is strengthened, leading to more positive emotional responses. However, if the eco-friendly functional experience is perceived as difficult to operate or inconvenient, consumers’ sense of identity and satisfaction are negatively impacted.

### 5.1 Theoretical contributions

Firstly, we employ a dual-path model to explore the impact of functional experiences on consumer satisfaction, and we identify emotional responses as the underlying mechanism through which functional experiences influence consumer satisfaction. In contrast to previous studies that rely solely on basic emotional responses to explain the effect of functional experiences on consumer satisfaction (Septianto et al., 2023), we incorporate emotional responses and perceived usability to develop a dual-path model integrating cognitively-oriented emotional responses (sense of efficiency) and affectively-oriented emotional responses (sense of identity). This theoretical framework allows us to validate that intelligent experience and eco-friendly experience elicit different emotional responses, leading to differential impacts on consumer satisfaction. Our findings provide a comprehensive perspective on the cognitive pathways of consumer satisfaction, contributing to a deeper understanding of the mechanisms underlying consumer satisfaction. This study offers new insights and methodologies for examining consumer satisfaction in the context of NEVs, enriching future academic research by serving as a valuable reference and promoting the advancement and refinement of consumer satisfaction theory.

Secondly, in classifying the functional experiences of new energy vehicles (NEVs), we identified two distinct functional attributes unique to NEVs and validated that both significantly impact consumer satisfaction. Unlike previous studies that analyzed the effect of singular, discrete functional experiences on consumer satisfaction (Lin et al., 2020; Li et al., 2023; Li and Shao, 2023), our research confirms the significant influence of intelligent experience and eco-friendly experience on consumer satisfaction. By systematically categorizing and studying functional experience variables, we not only extend existing research on the drivers of consumer satisfaction but also arrive at conclusions that differ from previous literature (Guo et al., 2021). Specifically, this study reveals the unique mechanisms through which intelligent experience and eco-friendly experience influence consumer satisfaction via cognitive and affective pathways, respectively, further advancing the application of the dual-path model in consumer behavior research.

Thirdly, in contrast to the perspective of previous research that solely considers perceived usability as a key determinant influencing consumer satisfaction (Devaraj et al., 2002), this study, taking into account the complexity of consumer emotional responses, specifies the boundary conditions for the impact of the two types of functional experiences in NEVs on emotional reactions. The research findings indicate that the influence of functional experiences on consumer satisfaction is moderated by perceived usability. It provides a novel perspective to gain a deeper understanding of how consumers under the influence of the two types of functional experiences elicit positive emotional responses, thereby further enhancing our comprehension of consumer satisfaction.

### 5.2 Practical implications

The conclusions of this study have important guiding implications for the practical application of new energy vehicle enterprises.

Firstly, with NEVs maintaining the global lead in sales for eight consecutive years and an increasing number of consumers choosing them, this study confirms that higher levels of intelligent and environmental attributes contribute to increased consumer satisfaction and the achievement of higher levels of industrialization for NEVs. In the current context of new energy vehicle enterprises facing technological bottlenecks, it is challenging to significantly enhance the intelligent and environmental attributes of NEVs in the short term to meet the diverse needs of customers. Therefore, new energy vehicle enterprises should analyze their positioning and consumer characteristics, focusing on the hedonic value and experience of NEVs based on the perceived value of the majority of consumers purchasing NEVs. This targeted approach in the production of NEVs, whether emphasizing intelligent attributes or environmental attributes, will contribute to technological innovation and application, helping new energy vehicle enterprises enhance consumer satisfaction.

Secondly, this study provides marketing practitioners with valuable insights for designing and managing marketing activities. While many enterprises see functional experiences as an opportunity to promote NEVs or introduce functional experience appeals in advertisements to boost sales (Li et al., 2023), this study points out that different types of functional experiences evoke different emotional response mechanisms. Therefore, marketing practitioners should not merely highlight functional experiences but should distinguish between types of functional experiences, ensuring that the benefits of new energy vehicle products conveyed in marketing activities or advertisements match the different emotional response mechanisms of consumers.

Thirdly, with the development of the Internet and the rise of online review platforms, online reviews have become increasingly important for service-oriented enterprises. Taking appropriate intervention measures in the face of negative word-of-mouth can enhance consumer satisfaction (Xiong and Shu, 2023). This study, conducted in the context of NEV driving experiences, acknowledges the positive significance of NEVs, promoting the implementation of the “dual carbon” strategy, enhancing consumer satisfaction, and encouraging sustainable consumption behaviors. By deeply analyzing the different mechanisms through which intelligent experience and eco-friendly experience affect consumer satisfaction, this research provides specific strategic guidance for NEV enterprises. For example, during the design and marketing process, emphasis should be placed on improving the usability and entertainment value of intelligent features while optimizing the practicality and convenience of eco-friendly features. Additionally, companies should prioritize online review feedback, promptly addressing and resolving negative consumer evaluations to enhance product and service experiences, thereby further boosting consumer trust and satisfaction in NEVs. These practical implications not only help NEV companies gain a competitive edge in the market but also provide crucial support for promoting green consumption and achieving sustainable development goals.

### 5.3 Research limitations and future prospects

Firstly, as an exploratory study, this research only tested Chinese consumers as the sample. Although the hypotheses proposed in this paper have been verified, the holistic thinking pattern of Chinese people has an impact on their cognitive processes. Due to space limitations, this study did not conduct empirical tests on Western consumers. It is speculated that if Western consumers are used as samples, due to their emphasis on individual and categorical analytical thinking rather than holistic and relational thinking, they may activate either the “rational” or “emotional” cognitive paths separately when facing different types of functional experiences, thus eliciting corresponding emotional responses (Bai et al., 2024). Future research can further examine this issue from the perspective of cognitive differences between Eastern and Western cultures.

Secondly, this study only focused on consumer satisfaction with NEVs and used new energy vehicle ratings as a measure of consumer satisfaction, which is consistent with previous research methods (Wu and Huo, 2023). Future research can use methods such as observation or in-depth interviews to gain a deeper understanding of consumers’ driving experiences with NEVs, such as whether the intelligent assisted driving of NEVs affects consumers’ perception of the value of these vehicles.

Thirdly, the text analysis method used in the study has limitations. Due to the lack of mature studies defining words related to intelligence and environmental aspects, and the absence of dictionaries containing words with different emotional polarities, it is challenging to quantify the degree of intelligent and environmental attributes of NEVs. Future research can develop dictionaries based on online text that include words with different emotional polarities and are more effective.

## Data Availability

The raw data supporting the conclusions of this article will be made available by the authors, without undue reservation.
